# Healthy dietary patterns, foods, and risk of glioma: A systematic review and meta-analysis of observational studies

**DOI:** 10.3389/fnut.2022.1077452

**Published:** 2023-01-04

**Authors:** Long Shu, Dan Yu, Fubi Jin

**Affiliations:** ^1^Department of Nutrition, Zhejiang Hospital, Hangzhou, Zhejiang, China; ^2^Department of Endocrinology, Zhejiang Hospital, Hangzhou, Zhejiang, China

**Keywords:** healthy dietary patterns, glioma, typical healthy foods, meta-analysis, observational studies, systematic review

## Abstract

**Background:**

Accumulating epidemiological evidence has shown the favorable associations between healthy dietary patterns and risk of glioma, although the results remain inconclusive.

**Objective:**

We therefore carried out a systematic review and meta-analysis to summarize the evidence from previous published studies, and to clarify the effects of healthy dietary patterns, typical healthy foods on glioma.

**Methods:**

PubMed, Web of Science, CNKI, and Wan fang data were searched from inception up to September 2022 for eligible studies. Two authors independently performed the literature search, study selection, data extraction, and quality assessment. Heterogeneity across studies was estimated using the Cochran’s *Q* test and *I*^2^ statistic. According to heterogeneity, the fixed-effects model or random-effects model was selected to obtain the relative risk (RR) of the merger. Subgroup analysis, sensitivity analysis and publication bias were also used for our analysis.

**Results:**

Twenty-four articles that met the selection criteria, involving 7,278 glioma cases and 2,143,528 participants, were included in our analysis. There was a reduced risk of glioma in the highest compared with the lowest categories of healthy dietary patterns (RR = 0.58; 95% CI: 0.44–0.77; *P* < 0.0001). Moreover, compared with the lowest intakes, the highest intakes of vegetables (RR = 0.84; 95% CI: 0.73–0.96; *P* = 0.012) and fruits (RR = 0.85; 95% CI: 0.72–1.00; *P* = 0.045) significantly reduce the risk of glioma. However, the intakes of fresh fish, nuts, whole grains, and dairy products showed no statistically significant associations with the risk of glioma (*P* > 0.05).

**Conclusion:**

Findings from this systematic review and meta-analysis indicate that higher intakes of healthy dietary patterns, vegetables, and fruits are significantly associated with the lower risk of glioma. Further studies, particularly with prospective design, are required to confirm our findings.

## Introduction

According to estimates from the International Agency for Research on Cancer (IARC) in 2020, the global incidence rate of brain cancer is 3.9 per 100,000 in males and 3.0 per 100,000 in females (1). Gliomas are the most common and devastating form of brain tumors in adults, accounting for approximately 80% of brain malignant tumors (2). In the United States, the latest data estimated that the average annual age-adjusted incidence rate of malignant brain and other central nervous system tumors was 7.08 per 100,000 in 2013–2017 (3). Considering the highly aggressive nature of glioma, a complete surgical resection is hard to achieve (4). Moreover, due to the high mortality rate, rapid onset and extremely poor prognosis, glioma has caused a severe disease burden for people (5). So far, exposure to high-dose ionizing radiation is the only clearly established environmental risk factor for glomia (6, 7). Thus, prioritizing the identification of the potential modifiable risk factors for glioma is of vital importance.

A growing body of evidence has suggested that dietary factors play the important role in the pathogenesis of glioma (8, 9). For decades, the majority of studies that reported the association between diet and glioma risk, have largely paid attention to individual nutrients, foods or food groups (10–13). In this regard, dietary intakes of vegetables, fruits, refined grains, processed meats and fish had been assessed (8, 14). For instance, Li found a protective effect of higher intakes of fruits against glioma in a previous meta-analysis (RR = 0.573, 95% CI: 0.346–0.947) (14). Likewise, a recent systematic review and meta- analysis published in 2022 by Zhang et al. higher intakes of processed meats, processed fish and grains were significantly associated with the increased risk of glioma (8). However, because of the complexity nature of dietary constituents and the potential interactions between foods and nutrients (15), these priori studies revealed the limited impact of diet on glioma. In this context, dietary pattern analyses, as new direction in nutritional epidemiology, have emerged to take the combined effects of foods and nutrients into account (16). More importantly, the results of dietary patterns analyses can be more readily translated into dietary guidelines, facilitating nutritional recommendations (17).

The considerable attentions in recent years have been focused on examining dietary patterns as a whole in relation to glioma risk (9). Up to date, several epidemiological studies have explored the associations between adherence to healthy dietary patterns and glioma risk (9, 18–26). Among these published studies, healthy dietary patterns, generally characterized by higher intakes of vegetables, fruits, whole grains, nuts, and legumes, included the Dietary Approaches to Stop Hypertension (DASH) diet, Mediterranean diet, the Alternative Healthy Eating Index (AHEI), and other dietary patterns that have similar dietary components (19). Nevertheless, the existing evidence of the associations between healthy dietary patterns and risk of glioma is still fairly controversial and inconclusive. Some of them have shown the significant protective effects of healthy dietary patterns against glioma (18–20), while other studies reported the positive or null associations (9, 21). A recent case-control study conducted by Mousavi et al. examining the association of diet with glioma concluded that adherence to the Mediterranean dietary pattern was associated with a lower likelihood of having glioma in Iranian adults (18). Similarly, Benisi-Kohansal et al. also showed that adherence to the DASH-style dietary pattern was inversely associated with glioma (highest tertile vs. lowest tertile: OR = 0.28, 95% CI = 0.13–0.57, *P* < 0.001) (19). However, in a hospital-based case-control study among Iranian adults, Malmir et al. failed to find any significant association between nutrient patterns defined through the use of *a posteriori* methods and odds of glioma (21). In contrast to the studies mentioned above, Kuan et al. found that there was a weak evidence for increased glioma risk associated with healthy dietary patterns (including DASH diet, AHEI diet, and Mediterranean diet), but these associations were generally null after excluding the first 5 years of follow-up (9). Notably, a recent meta-analysis of 33 observational studies on dietary factors and risk of glioma showed that higher intakes of tea, total vegetables, and orange vegetables might reduce the risk of glioma, while higher intakes of grains, processed meats, and processed fish might increase the risk of glioma (8). But, the above mentioned meta-analysis on the association between diet and glioma has mainly focused on 12 food groups (e.g., total vegetables, fruits, grains, processed meats, etc.), and has a methodological limitation. Furthermore, to our knowledge, there has been no systematic review and meta-analysis to assess the associations between whole dietary patterns and risk of glioma. In view of these literature gaps, the purpose of this systematic review and meta-analysis was to systematically review and summarize the evidence from previous studies published up to September 30, 2022 and to explore the associations between healthy dietary patterns, typical healthy foods, and risk of glioma using meta-analysis.

## Materials and methods

This systematic review and meta-analysis was performed in accordance with the Preferred Reporting Items for Systematic Reviews and Meta-Analyses (PRISMA) 2020 guidelines (27). However, the protocol for this review was not registered at PROSPERO.

### Literature search strategy

A comprehensive literature search was performed for pertinent articles published in English or Chinese using the databases of PubMed, Web of Science, CNKI, and Wan Fang Data up to September 2022, with the following search terms: (“glioma”[all fields] OR “gliblastoma” [all fields] OR “brain cancer”[all fields] OR “brain tumor”[all fields]) AND (“diet”[all fields] OR “food”[all fields] OR “dietary pattern” [all fields] OR “food pattern”[all fields]). No publication date restrictions were applied in the retrieved process. Moreover, reference lists of all retrieved studies and the published review articles were also manually searched to identify any other studies that were not found in the initial database search.

### Studies included criteria

Titles and abstracts of all retrieved articles were assessed by two independent authors (LS and DY). Any inconsistencies were resolved by discussion with a third author until consensus. After all authors agreed on the relevant articles, the full-text versions of articles were reviewed against inclusion and exclusion criteria for this meta-analysis. Studies were included in this systematic review and meta-analysis if they met the following inclusion criteria: (1) original studies with observational design (including case-control, nested case-control or prospective/retrospective cohort); (2) the study was published in the English or Chinese languages; (3) the exposure of interest was healthy dietary patterns and typical healthy foods; (4) the outcome of interest was glioma in adults; (5) studies that reported risk estimates [relative risks (RRs), odds ratios (ORs), and hazards ratios (HRs)] of glioma and their corresponding 95% CIs were provided (or sufficient data to calculate them); (6) If the data in original publication lacked sufficient detail, the corresponding author of the study was contacted for additional information by email. Studies were excluded based on the following criteria: (1) written in a language other than English or Chinese; (2) not performed on humans; (3) non-observational study, i.e., review articles, conference abstracts, case reports, editorials, and letters to the editor; (4) lack of sufficient data to obtain RR/HR/OR and 95% CI.

### Data extraction

Two authors (LS and DY) independently assessed the eligible studies. Among the selected studies, the following information was extracted, including the first author’s last name, year of publication, location, study design, age range/mean age for cases and participants, number of cases and controls or participants, methods used to identify dietary pattern, confounders adjusted for, and the OR, RR, or HR estimates with corresponding 95% CI for the highest vs. lowest categories of healthy dietary patterns or typical healthy foods.

### Quality assessment

The Newcastle-Ottawa Scale (NOS) was used to evaluate the quality of the included non-randomized studies in meta-analyses (28). This scale consists of 3 main domains: selected population (4 points), comparability of groups (2 points), and assessment of either the outcome/exposure of interest for cohort or case-control studies (3 points), with a maximum score of 9 points. Studies with a NOS score of 7 or above were considered as of high quality (28). The quality assessment was carried out independently by two authors, and disagreements were resolved by discussion or in consultation with the third author (FJ).

### Statistical analysis

All statistical analyses were conducted using STATA software, version 12.0 (StataCorp, College Station, TX, USA). Given the incidence of glioma was relatively low, ORs and HRs were directly treated as RRs (29). Log-transformed RRs with corresponding standard errors (SEs) were used to estimate the associations between healthy dietary patterns, typical healthy foods and glioma risk. The Cochran’s Q test and *I*^2^ statistic were used to evaluate the potential sources of heterogeneity in all included studies. A *P*-value of *Q*-test ≤ 0.10 or *I*^2^ scores ≥ 50% indicated a presence of between-study heterogeneity, and the random-effects model by DerSimonian and Laird method was used to combine the effect size and 95% CI. Otherwise, the fixed-effects model was used (30). If the results showed significant heterogeneity, potential sources of heterogeneity were explored by using sensitivity and subgroup analyses. Subgroup analyses were performed to determine whether the potential sources of heterogeneity across studies came from the study design (cohort/case-control studies), sample size (<1,000 vs. ≥1,000), country (United States/Asian countries), the methods used to determine dietary patterns (*a priori* vs. *a posteriori*), and the study quality (≥7 points and <7 points). Publication bias was evaluated visually with funnel plots and with the Begg’s or Egger’s tests (31). Sensitivity analyses were performed, excluding one study at a time to clarify whether the results were robust or sensitive to the influence of an individual study or a group of studies. A two-tailed *P*-value < 0.05 was considered of statistical significance.

## Results

### Overview of included studies for the systematic review

**FIGURE 1:**
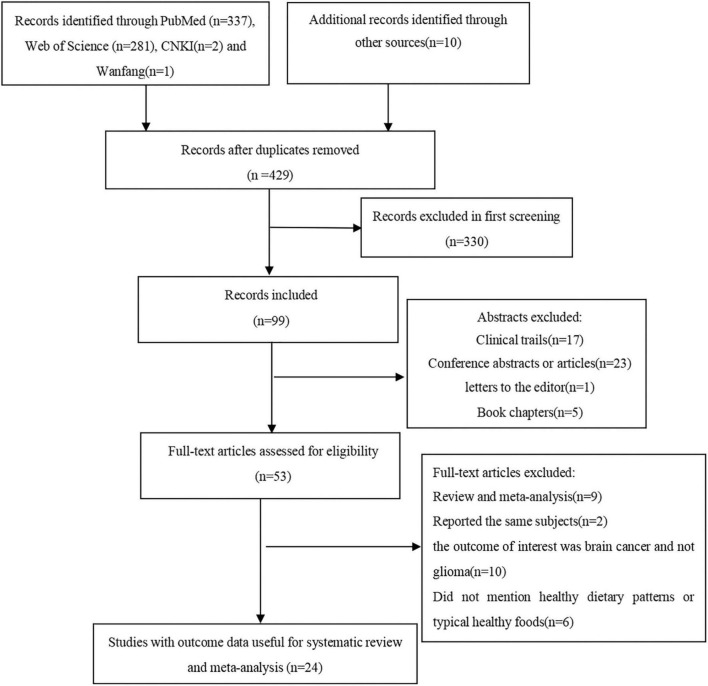
Flow chart of article screening and selection process.

Figure 1 shows the flow chart of article screening and selection process. We identified 631 potentially relevant articles by searching PubMed, Web of Science, CNKI, and Wan fang data. After excluding duplicates between four databases, 429 articles remained. Reviewing titles and abstracts led to the exclusion of 376 studies because they did not report the associations between dietary patterns or foods and risk of glioma, and 53 articles remained for full-text review. Of the remaining 53 articles, 29 articles were excluded for the following reasons: systematic reviews (*n* = 9); the outcome of interest was brain tumor and not glioma (*n* = 10); reported the same participants (*n* = 2); did not mention healthy dietary patterns or typical healthy foods (*n* = 6); animal studies (*n* = 2). Ultimately, 24 articles were eligible to be included in this systematic review and meta-analysis. Among these articles, 10 articles reported the association between healthy dietary patterns and risk of glioma, 13 articles reported the association between vegetables intake and risk of glioma, 16 articles reported the association between fruits intake and risk of glioma.

### Study characteristics

The characteristics of the included studies on the associations between healthy dietary patterns, foods, and glioma risk are shown in Table 1. Twenty-four articles (reporting 28 studies) with 2,143,528 participants and 7,278 cases of glioma were included in our analysis. The majority of the included studies were case-control in design (11, 18–26, 32–41), and four of them were cohort design (9, 10, 42, 43). Publication dates of these studies varied between 1987 and 2022. The age of participants ranged from ages 18 to above. Nine of the included studies were conducted in Iran (18–26), three in China (38, 40), eight in the United States (9–11, 34, 41–43), one in Germany (35), one in France (32), one in Canada (36), one in Australia (37), one in the United Kingdom (9), and a multi-center study was conducted in six different locations of developed countries (33). Among the included studies, 12 studies reported the association between healthy dietary patterns and glioma risk (9, 18–26). Three of the included studies derived dietary patterns through *a posteriori* method (20–22), and nine reported the associations for dietary scores defined *a priori* approach (9, 18, 19, 23–26). All the included studies used FFQs to assess dietary intake. Adjustment-variables were mostly age, sex, energy intake, physical activity, family history of glioma, marital status, alcohol consumption, and body mass index. Overall, 16 studies were classified as of high quality (9–11, 21, 22, 25, 32–37, 40–43), and the remaining eight studies were of medium quality (18–20, 23, 24, 26, 38, 39).

**TABLE 1:** Characteristics of included studies on the association between healthy dietary patterns, foods, and risk of glioma (–2022).

References	Location	Study design	Total number of subjects	Age	Method used to identify dietary pattern	Adjustment or matched for in analyses	RR (95% CI) for highest vs. lowest category
Kuan et al. (9)	UK, USA	Cohort	692,176 (1,173 cases); 470,780 (1,005 cases); 99,148 (135 cases)	50–74 years	Priori	Height, body mass index, smoking, alcohol intake, level of educational attainment, region of residence, parity, oral contraceptive use, and use of menopausal hormones for women.	AHEI: 1.14 (1.01–1.28); aMED: 1.24 (1.05–1.46); DASH: 1.19 (1.05–1.34); 1.03 (1.0–1.06) for total fruit; 1.03 (0.99–1.06) for total vegetables; 1.03 (0.97–1.08) for nuts; 1.03 (0.98–1.08) for grains; 0.97 (0.84–1.12) for fish; 1.00 (0.98–1.02) for dairy products
Holick et al. (10)	USA	Cohort	289,915 (296 cases)	30–75 years	NA	Age and total calorie intake	1.41 (0.95–2.10) for fruits; 1.17 (0.78–1.75) for vegetables
Chen et al. (11)	USA	Case-control	236 cases 449 controls	≥21 years	NA	Age, age squared, sex, TEI, respondent type, education level, family history, and farming experience.	0.5 (0.3–1.0) for vegetables 1.0 (0.6–1.7) for fruits 0.60 (0.30–1.20) for fish 1.80 (1.00–3.30) for dairy products
Mousavi et al. (18)	Iran	Case-control	128 cases 256 controls	20–75 years	Priori	Age, sex, energy intake, physical activity, family history of cancer, family history of glioma, marital status, education, high-risk job, high-risk living area, cell phone usage time, supplement use, history of exposure to the radiographic X-ray, history of head trauma, history of allergy, history of hypertension, smoking status, exposure to chemicals, medication use, personal hair dye use, frequent fried food intake, frequent use of barbecue, canned foods, microwave, refined grains, tea, coffee, egg, and BMI.	Mediterranean dietary pattern: 0.36 (0.16–0.78)
Benisi-Kohansal et al. (19)	Iran	Case-control	128 cases 256 controls	20–75 years	Prior	Age, sex, energy intake, physical activity, family history of cancer, family history of glioma, marital status, education, high-risk occupation, high-risk residential area, duration of cell phone use, supplement use, history of exposure to the radiographic X-ray, history of head trauma, history of allergy, history of hypertension, smoking status, exposure to chemicals, drug use, personal hair dye use, frequent fried food intake, frequent use of barbecue, canned foods, microwave, and BMI.	DASH diet: 0.28 (0.13–0.57) Nuts intake: 0.24 (0.11–0.55) Dairy products intake: 0.56 (0.26–1.24) Whole grain intake: 1.06 (0.48–2.30)
Mousavi et al. (20)	Iran	Case-control	128 cases 256 controls	20–75 years	Posteriori	Age, sex, energy intake, physical activity, family history of glioma, marital status, high-risk job, high-risk living area, duration of cell phone usage, supplement use, history of exposure to the radiographic X-ray, history of dental photography, history of head trauma, smoking status, exposure to chemicals, personal hair dye use, frequent use of fried food, and microwave.	hPDI: 0.32 (0.18–0.57)
Malmir et al. (21)	Iran	Case-control	128 cases 256 controls	43.1 years	Posteriori	Age, sex, energy intake, physical activity, family history of cancers, family history of glioma, marital status, education, high-risk occupation, high-risk residential area, duration of cell phone use, supplement use, history of exposure to the radiographic X-ray, history of head trauma, history of allergy, history of hypertension, smoking status, exposure to chemicals, drug use, personal hair dye, frequent fried food intake, frequent use of barbecue, canned foods, microwave, and BMI.	Nutrient pattern1: 0.71 (0.35–1.42) Nuts intake: 0.34 (0.15–0.76)
Ebrahimpour-Koujan et al. (22)	Iran	Case-control	128 cases 256 controls	20–75 years	Posteriori	Age, gender, physical activity, family history of cancer, family history of glioma, marital status, education, high-risk job, high-risk living area, duration of cell phone use, supplement use, history of exposure to the radiographic X-ray, history of head trauma, history of allergy, history of hypertension, smoking status, exposure to chemicals, medication use, personal hair dye use, frequent fried food intake, frequent use of barbecue, canned foods and microwave, dietary intakes of vitamin A, vitamin C, vitamin D, vitamin E, vitamin B12, vitamin B6, zinc, copper, potassium, calcium, selenium, total fiber, salt, cholesterol, methionine, and BMI.	LCD: 0.32 (0.12–0.81)
Sadeghi et al. (23)	Iran	Case-control	128 cases 256 controls	43.1 years	Priori	Energy intake, family history of glioma, marital status, high-risk occupation, high-risk residential area, supplement use, history of exposure to radiographic X-rays, history of head trauma, smoking, drug use, personal hair dye use, frequent fried food intake and frequent use of a microwave, BMI.	AHEI: 0.26 (0.12–0.56)
Soltani et al. (24)	Iran	Case-control	128 cases 256 controls	20–75 years	Priori	Age, sex, energy intake, physical activity, family history of cancer, family history of glioma, marital status, education, high-risk occupation, high-risk residential area, duration of cell phone use, supplement use, drug use, history of exposure to the radiographic X-ray, history of head trauma, history of allergy, history of hypertension, smoking status, exposure to chemicals, personal hair dye use, frequent fried food intake, frequent use of barbecue, canned foods, microwave, and BMI.	MIND diet: 0.39 (0.18–0.84)
Ebrahimpour-Koujan et al. (25)	Iran	Case-control	128 cases 256 controls	20–75 years	Priori	Age, sex, marital status, education, high-risk occupation, high-risk residential area, duration of cell phone use, supplement use, drug use, smoking status, exposure to chemicals, personal hair dye use, family history of cancer, family history of glioma, history of exposure to the radiographic X-ray, history of head trauma, history of allergy, history of hypertension, frequent fried food intake, frequent use of barbecue, canned foods, and microwave.	HLS: 0.29 (0.12–0.70)
Rigi et al. (26)	Iran	Case-control	128 cases 256 controls	20–75 years	Priori	Age, sex, and energy intake, physical activity, family history of cancer, family history of glioma, marital status, education, high-risk residential area, duration of cell phone use, supplement use, history of exposure to the radiographic X-ray, history of head trauma, history of allergy, history of hypertension, smoking status, exposure to chemicals, drug use, personal hair dye use, frequent fried food intake, frequent use of barbecue, canned foods and microwave, red and processed meat, fish, tea and coffee and, sugar-sweetened beverage, egg, total fat, dietary fiber, cholesterol, folate, selenium	DPI: 0.43 (0.19–0.97)
Cabaniols et al. (32)	France	Case-control	79 cases 83 controls	≥18 years	NA	Age and sex	0.85 (0.49–1.47) for fruit
Terry et al. (33)	Germany, France, Canada, Sweden, Australia, USA	Case-control	1,185 cases 2,486 controls	20–80 years	NA	Age, sex, center, and the following food groups: leafy green vegetables, yellow-orange vegetables, cured meat, non-cured meat, fresh fish, dairy eggs, grains, and citrus fruit.	1.4 (1.1–1.8) for fruit 1.17 (0.78–1.75) for vegetables 0.90 (0.70–1.10) for fish
Blowers et al. (34)	USA	Case-control	94 cases 91 controls	25–74 years	NA	Matched the patient on age (within 5 years) and race (black or white).	1.3 (0.5–3.0) for fruit 1.3 (0.5–3.2) for vegetables 0.40 (0.20–1.10) for fish 0.90 (0.40–2.20) for nuts 2.10 (0.80–5.20) for dairy products
Boeing et al. (35)	Germany	Case-control	115 cases 418 controls	20–75 years	NA	Age, gender, tobacco-smoking, and alcohol consumption	1.1 (0.6–1.9) for fruit 0.9 (0.5–1.7) for vegetables 0.70 (0.40–1.40) for fish
Burch et al. (36)	Canada	Case-control	215 cases 228 controls	25–80 years	NA	Matched to each case on the basis of sex, area of residence, marital status, year of birth (within 5 years), date of diagnosis (within 1 year) for live cases, and date of death (within 1 year) for dead cases.	1.33 (0.56–3.16) for vegetables 0.58 (0.34–1.00) for fruit
Giles et al. (37)	Australia	Case-control	409 cases 409 controls	20–70 years	NA	Alcohol and tobacco	Men: 1.01 (0.62–1.63) for vegetables 1.51 (0.95–2.39) for fruits 1.29 (0.79–2.12) for fish 1.36 (0.76–2.35) for nuts 0.66 (0.37–1.25) for whole grain Women: 0.53 (0.29–0.95) for vegetables 0.69 (0.36–1.31) for fruits 0.63 (0.32–1.26) for fish 0.92 (0.49–1.75) for nuts 0.92 (0.49–1.75) for whole grain
Hu et al. (38)	China	Case-control	218 cases 436 controls	Mean: 39.6 years	NA	Income, education, number of years, drinking alcohol, and occupational exposures	0.51 (0.29–0.89) for vegetables 0.28 (0.16–0.51) for fruits
Hu et al. (39)	China	Case-control	73 cases 311 controls	20–74 years	NA	Income, education, cigarette smoking, alcohol intake, selected occupational exposures, and total energy intake	0.29 (0.1–0.7) for vegetables 0.15 (0.1–0.4) for fruits 0.38 (0.2–0.9) for fresh fish
Xu et al. (40)	China	Case-control	86 cases 258 controls	≥18 years	NA	Age, sex, and economic conditions	Men: 0.84 (0.73–0.96) for vegetables 0.85 (0.77–0.95) for fruits Women: 0.81 (0.73–0.89) for vegetables 0.70 (0.54–0.91) for fruit
Mills et al. (42)	USA	Cohort	34,000 (20 cases)	≥25 years	NA	Age and sex	0.85 (0.28–2.60) for fruit
Preston-Martin et al. (41)	USA	Case-control	202 cases 202 controls	25–69 years	NA	NA	0.8 (0.4–1.6) for fruit
Dubrow et al. (43)	USA	Cohort	545,770 (585 cases)	50–71 years	NA	Sex, age, race, energy intake, education, height, and history of cancer at baseline	1.17 (0.89–1.53) for vegetables 1.16 (0.89–1.52) for fruits

BMI, body mass index; USA, United States; UK, United Kingdom; AHEI, alternative healthy eating index; aMED, alternate Mediterranean diet; DASH, dietary approaches to stop hypertension; hPDI, healthy plant-based diet; LCD, low carbohydrate diet; MIND, Mediterranean-DASH diet intervention for neurodegenerative delay diet; HLS, healthy lifestyle score; DPI, dietary phytochemical index.

### Healthy dietary patterns and risk of glioma

**FIGURE 2:**
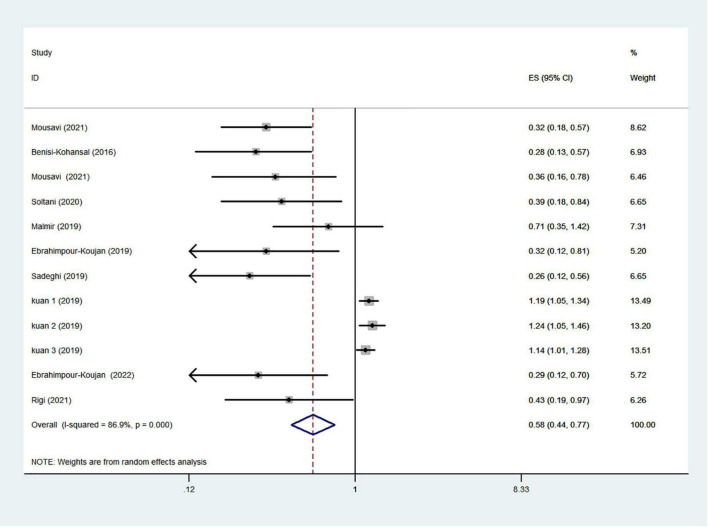
Healthy dietary patterns and risk of glioma.

The healthy dietary patterns are characterized by higher intakes of vegetables, fruits, fish, nuts, legume, olive oil, whole grains, and lower intakes of refined grains, red meat, high-fat dairy products. Ten articles reporting 12 original studies were included to assess the associations between healthy dietary patterns and glioma. Figure 2 showed the evidence of a decreased risk of glioma in the highest compared with lowest categories of healthy dietary patterns (RR = 0.58; 95% CI: 0.44–0.77; *P* < 0.0001). The heterogeneity among the included studies was apparent (*P* < 0.00001; *I*^2^ = 86.9%), and hence the effect was assessed using the random-effects model. There was an asymmetry in the funnel plot, and the Egger’s test (*P* < 0.0001) revealed a possible publication bias. Consistently, on the basis of the trim and fill algorithm, the adjusted value showed an inverse association between healthy dietary patterns and risk of glioma. Comparing the adjusted value (0.543) with the original estimate (0.581) indicates a small contribution of the study effect to the original results. The results from sensitivity analysis revealed that excluding an individual study did not change the significance of the results, indicating that the combined results are stable (Supplementary Figure 1).

### Typical healthy foods and risk of glioma

**FIGURE 3:**
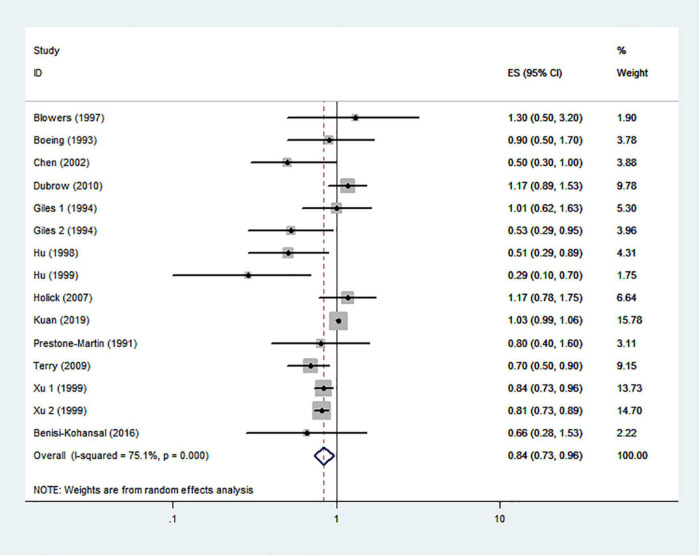
Vegetables intake and risk of glioma.

**FIGURE 4:**
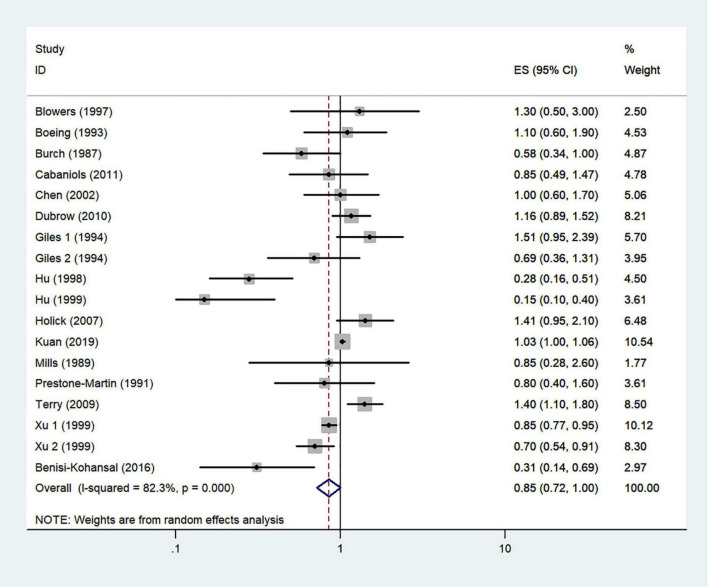
Fruits intake and risk of glioma.

**FIGURE 5:**
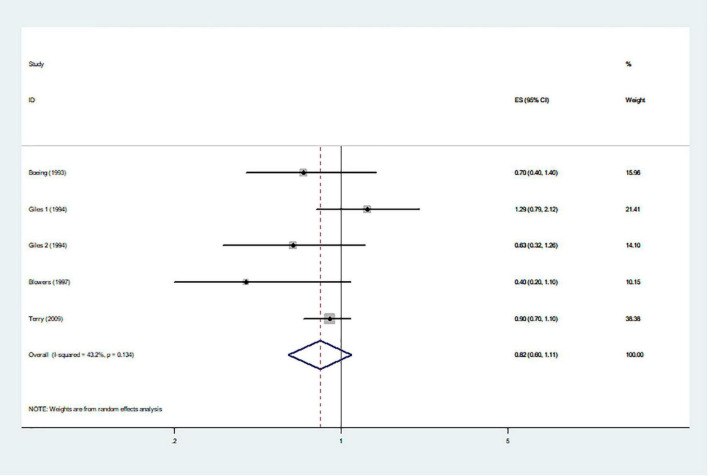
Fresh fish and risk of glioma.

**FIGURE 6:**
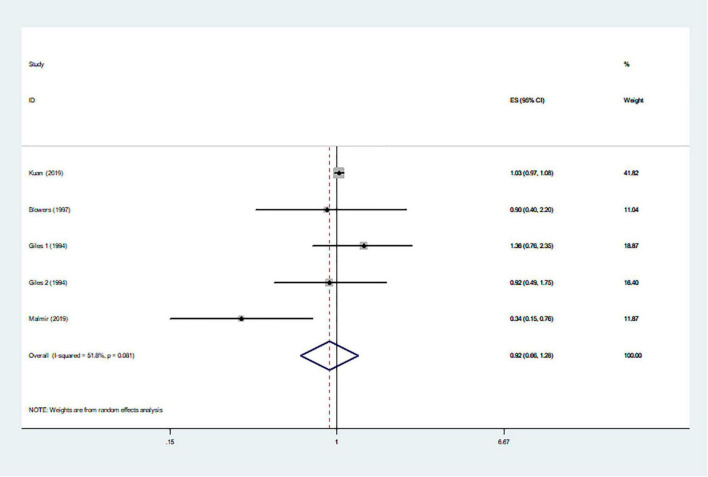
Nuts intake and risk of glioma.

**FIGURE 7:**
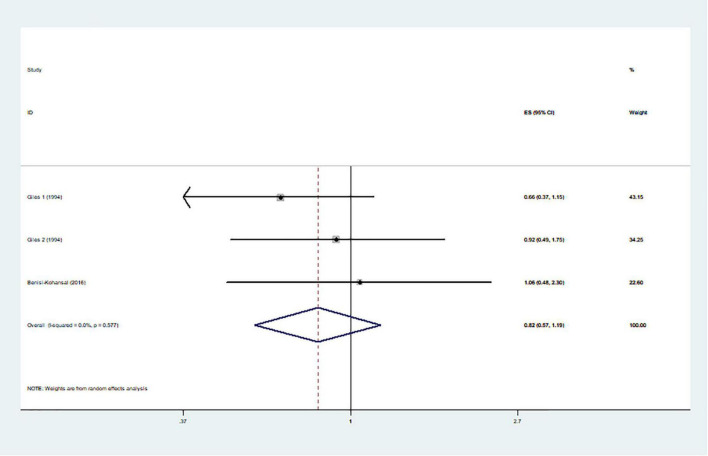
Whole grains intake and risk of glioma.

**FIGURE 8:**
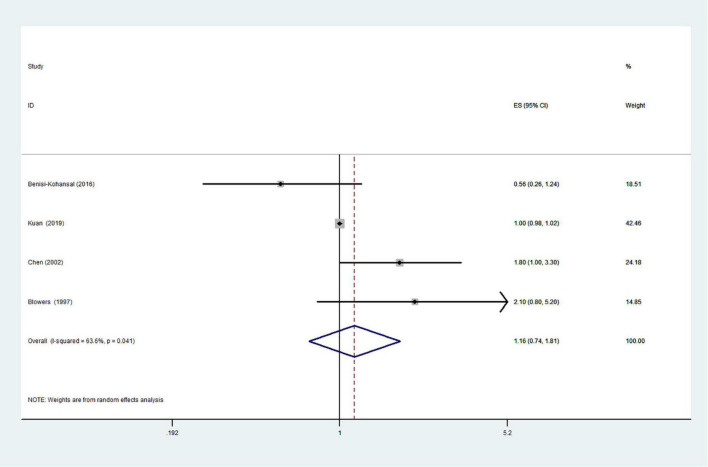
Dairy products intake and risk of glioma.

The effects of higher intakes of typical healthy foods on glioma risk are shown in Figures 3–8. Pooled results from 13 articles (including 15 original studies) showed an inverse association between vegetables intake and risk of glioma (RR = 0.84; 95% CI: 0.73–0.96; *P* = 0.012) (Figure 3). Data from these studies were assessed using random-effects model, and there was significant heterogeneity (*I*^2^ = 73.1%, *P* < 0.0001). Sixteen articles reporting eighteen original studies were included, and Figure 4 showed that there was evidence of a reduced risk of glioma in the highest intake of fruits compared with the lowest intake (RR = 0.85; 95% CI: 0.72–1.00; *P* = 0.014). A random-effects model was used to assess the data, and there was evidence of heterogeneity (*I*^2^ = 82.3%, *P* < 0.0001). However, in pooled analyses of five studies, high intake of fresh fish was not associated with the risk of gioma (RR = 0.82; 95% CI: 0.60–1.11; *P* = 0.197), and there was moderate between-study heterogeneity (*I*^2^ = 43.2%, *P* = 0.134) (Figure 5). Likewise, the pooled results of nuts (Figure 6), whole grains (Figure 7), and dairy products (Figure 8) showed that they were no related the risk of glioma.

### Subgroup analyses

Significant heterogeneity was observed in healthy dietary patterns, vegetables, fruits and nuts intake and glioma risk. Thus, further subgroup analyses were carried out. For healthy dietary patterns (Table 2), subgroup analyses was stratified basing on study design, methods used to determine dietary patterns, country, study quality, and comparison. Results showed that healthy dietary patterns was significantly associated with glioma risk in studies with case-control design, from Iran and with medium-quality score (pooled RR = 0.36, 95% CI: 0.28–0.46; *I*^2^ = 0%). Besides, in T3 vs. T1 comparison studies, there was no evidence of heterogeneity between studies (*P* = 0.703, *I*^2^ = 0%), and a significantly decreased risk of glioma was shown (RR = 0.37; 95% CI: 0.29–0.49). For other healthy foods intake (Table 3), subgroup analyses was stratified by study design, country, study quality and sample size. In the study design, vegetables intake was a significant inverse association in the case-control subgroup (RR = 0.77; 95% CI: 0.68–0.86), and a marginally positive association in the cohort subgroup (RR = 1.03; 95% CI: 1.00–1.07). In addition, fruits intake had significant association with risk of glioma in case-control studies (RR = 0.73; 95% CI: 0.57–0.94), despite significant heterogeneity (*I*^2^ = 82.1%, *P* < 0.0.001). For the study country, vegetables intake was statistically significant in Asian countries (RR = 0.77; 95% CI: 0.66–0.90), where moderate heterogeneity (*I*^2^ = 45.7%, *P* = 0.118). For study quality, vegetables and fruits intake were statistically significant in the studies with the study quality score < 7 (vegetables: RR = 0.49, 95% CI: 0.32–0.75; fruits: RR = 0.32, 95% CI: 0.16–0.62). However, the heterogeneity was apparent for fruits intake (*P* = 0.009; *I*^2^ = 73.9%). Similarly, for sample size, vegetables intake was statistically significant in the studies with sample size < 1,000 (RR = 0.77, 95% CI: 0.68–0.88). For fruits intake, there was evidence of heterogeneity (*P* < 0.0001; *I*^2^ = 78.0%), and a significant decrease in the risk of glioma was shown in the studies with sample size < 1,000 (RR = 0.68; 95% CI: 0.53–0.89). For fresh fish and whole grains intakes, because of the relatively small number of studies and limited reporting of subgroup results, we were unable to explore all potential sources of heterogeneity. In general, subgroup analyses revealed that several factors, e.g., study design, study quality, and methods used to determine dietary patterns are potential sources of heterogeneity.

**TABLE 2:** Subgroup analyses of healthy dietary patterns and glioma risk.

Dietary patterns	Subgroup	Number	RR (95% CI)	*I*^2^ (%)	*P*_for heterogeneity_
Healthy dietary patterns	Study design				
	Case-control	9	0.36 (0.28–0.46)	0.0	0.712
	Cohort	3	1.18 (1.09–1.27)	0.0	0.708
	Methods used to determine healthy dietary patterns				
	Priori	9	0.67 (0.50–0.88)	86.5	<0.0001
	Posteriori	3	0.42 (0.25–0.72)	39.8	0.190
	Country				
	Iran	9	0.36 (0.28–0.46)	0.0	0.712
	Western countries	3	1.18 (1.09–1.27)	0.0	0.708
	Study quality				
	≥7	3	1.18 (1.09–1.27)	0.0	0.708
	<7	9	0.36 (0.28–0.46)	0.0	0.712
	Comparison				
	Q4 vs. Q1	4	1.09 (0.89–1.33)	80.5	0.002
	T3 vs. T1	8	0.37 (0.29–0.49)	0.0	0.703

Q4, quartile 4; Q1, quartile 1; T3, tertile 3; T1, tertile 1.

**TABLE 3:** Subgroup analyses of healthy foods and glioma risk.

Dietary factors	Subgroup	Number	RR (95% CI)	*I*^2^ (%)	*P*_for heterogeneity_
Vegetables	Study design				
	Case-control	12	0.77 (0.68–0.86)	23.9	0.209
	Cohort	3	1.03 (1.00–1.07)	0.0	0.548
	Country				
	Asian countries	5	0.77 (0.66–0.90)	45.7	0.118
	Western countries	10	0.91 (0.77–1.08)	52.6	0.025
	Study quality				
	≥7	12	0.89 (0.78–1.01)	74.9	<0.001
	<7	3	0.49 (0.32–0.75)	0.0	0.447
	Sample size				
	≥1,000	4	1.00 (0.83–1.20)	61.8	0.049
	<1,000	11	0.77 (0.68–0.88)	26.9	0.188
Fruits	Study design				
	Case-control	14	0.73 (0.57–0.94)	82.1	<0.0001
	Cohort	4	1.05 (0.97–1.13)	7.4	0.356
	Country				
	Asian countries	5	0.42 (0.25–0.70)	90.5	<0.0001
	Western countries	13	1.09 (0.95–1.24)	36.5	0.091
	Study quality				
	≥7	14	1.00 (0.88–1.14)	66.7	<0.0001
	<7	4	0.32 (0.16–0.62)	73.9	0.009
	Sample size				
	≥1,000	5	1.17 (0.99–1.39)	55.6	0.061
	<1,000	13	0.68 (0.53–0.89)	78.0	<0.0001
Nuts	Study design				
	Case-control	4	0.82 (0.47–1.44)	60.4	0.055
	Cohort	1	1.03 (0.98–1.09)	–	–
	Country				
	Asian countries	1	0.34 (0.15–0.77)	–	–
	Western countries	4	1.03 (0.98–1.09)	0.0	0.766
	Sample size				
	≥1,000	1	1.03 (0.98–1.09)	–	–
	<1,000	4	0.82 (0.47–1.44)	60.4	0.055
Dairy products	Study design				
	Case-control	3	1.28 (0.58–2.85)	69.8	0.037
	Cohort	1	1.00 (0.98–1.02)	–	–
	Country				
	Asian countries	1	0.56 (0.26–1.22)	–	–
	Western countries	3	1.38 (0.82–2.30)	67.4	0.047
	Study quality				
	≥7	3	1.38 (0.82–2.30)	67.4	0.047
	<7	1	0.56 (0.26–1.22)	–	–
	Sample size				
	≥1,000	1	1.00 (0.98–1.02)	–	–
	<1,000	3	1.28 (0.58–2.85)	69.8	0.037

### Sensitivity analysis and publication bias

The effect of individual studies on the pooled RR was evaluated by repeating the meat-analysis after eliminating each study in turn. The results showed a slight change in the relationship between healthy dietary patterns and glioma, when Kuan et al.’s study was excluded. Besides, no individual study had excessive influence on the association of typical healthy foods intake and glioma, when we removed a single study at a time. Hence, the results of this meta-analysis were relatively stable (Supplementary Figure 1). The funnel plots and asymmetry tests showed publication bias for the healthy dietary patterns (Begg’s test *P* = 0.271; Egger’s test *P* < 0.01). Thus, we conducted the trim and fill analysis to adjust the pooled effect, and found that the results remained unchanged. Besides, for other healthy foods, the visual inspection of funnel plots revealed little evidence of asymmetry (not shown) and therefore little evidence of publication bias (highest compared with lowest categories: Begg’s test, vegetables intake: *P* = 0.843; fruits intake: *P* = 0.120; fresh fish intake: *P* = 0.086; nuts intake: *P* = 0.462; whole grains: *P* = 0.296; dairy products: *P* = 1.00).

### Quality assessment

The quality of included non-randomized studies based on NOS criteria is shown in Table 4. When included studies received a score of seven or higher, they would be deemed to be of relatively higher quality (9–11, 21, 22, 25, 32–37, 40–43). Moreover, the remaining eight studies were identified as medium-quality studies (18–20, 23, 24, 26, 38, 39).

**TABLE 4:** Healthy dietary patterns, typical healthy foods, and glioma: Assessment of study quality.

References	Selection				Comparability		Outcome			
	1	2	3	4	5A	5B	6	7	8	Score
**Cohort**										
Holick et al. (10)	*	*		*	*	*	*	*		8
Mills et al. (42)	*	*	*	*	*		*	*	*	8
Dubrow et al. (43)	*	*	*	*	*	*	*	*	*	7
Kuan et al. (9)	*	*	*	*	*	*	*	*	*	8
**Case-control**	*	*	*	*	*		*	*		
Burch et al. (36)	*	*	*	*	*	*	*	*	*	7
Preston-Martin et al. (41)	*	*	*	*	*		*	*		7
Boeing et al. (35)	*	*	*		*		*	*	*	7
Giles et al. (37)	*	*	*		*		*	*	*	7
Blowers et al. (34)	*			*	*	*	*	*	*	7
Hu et al. (38)	*			*	*	*	*	*		6
Hu et al. (39)	*			*	*	*	*	*		6
Xu et al. (40)	*			*	*	*	*	*	*	7
Chen et al. (11)	*			*	*	*	*	*	*	7
Terry et al. (33)	*			*	*	*	*	*	*	7
Cabaniols et al. (32)	*			*	*	*	*	*	*	7
Benisi-Kohansal et al. (19)	*			*	*	*	*	*		6
Mousavi et al. (18)	*			*	*	*	*	*		6
Mousavi et al. (20)	*			*	*	*	*	*		6
Malmir et al. (21)	*			*	*	*	*	*	*	7
Soltani et al. (24)	*			*	*	*	*	*		6
Ebrahimpour-Koujan et al. (22)	*			*	*	*	*	*	*	7
Sadeghi et al. (23)	*			*	*	*	*	*		6
Ebrahimpour-Koujan et al. (25)	*			*	*	*	*	*	*	7
Rigi et al. (26)	*			*	*	*	*	*		6

*For case-control studies, 1, indicates cases independently validated; 2, cases are representative of population; 3, community controls; 4, controls have no history of blood pressure disease; 5A, study controls for age; 5B, study controls for additional factor(s); 6, ascertainment of exposure by blinded interview or record; 7, same method of ascertainment used for cases and controls; and 8, non-response rate the same for cases and controls. For cohort studies, 1, indicates exposed cohort truly representative; 2, non-exposed cohort drawn from the same community; 3, ascertainment of exposure; 4, outcome of interest not present at start; 5A, cohorts comparable on basis of age; 5B, cohorts comparable on other factor(s); 6, quality of outcome assessment; 7, follow-up long enough for outcomes to occur; 8, complete accounting for cohorts.

## Discussion

To the best of our knowledge, this is the first systematic review and meta-analysis to explore the associations between healthy dietary patterns, typical healthy foods and the risk of glioma. Based on 24 articles on healthy dietary patterns, typical healthy foods and glioma published from 1987 to 2022, involving 7,278 glioma cases and 2,143,528 participants, our meta-analysis results showed the significant inverse associations between healthy dietary patterns, vegetables and fruits intakes and risk of glioma, with substantial heterogeneity between studies. Meanwhile, other healthy foods including fresh fish, whole grains, nuts and dairy products had no significant effects on glioma. Subgroup analyses revealed that these associations depend on the study design, study quality, country, and methods used to identify dietary patterns. Collectively, our findings provide further evidence for an inverse association between healthy dietary patterns and glioma risk, and support the adoption of this pattern for the prevention of glioma.

In 2020, GLOBCAN estimated that the overall age-standardized incidence rate of brain cancer was 3.9 and 3.0 per 100,000 for males and females, respectively (1). Despite the relatively low incidence, glioma, the most common primary brain cancer, is significantly associated with high mortality and poor prognosis (6). In addition, it is reported that more than 97% of glioma patients die within 5 years after diagnosis (44). Thus, finding the contributing factors to the incidence and development of glioma is of great importance. More recently, greater attention has been paid to the influence of diet as a whole on glioma. Dietary patterns can better reflect the eating habits of the study population. To date, numerous epidemiological studies have examined the associations between healthy dietary patterns and glioma risk (9, 18, 19, 21), but the results remain inconsistent. Currently, the healthy dietary patterns such as Mediterranean, AHEI and DASH diets, characterized by high intake of fruits, vegetables, whole grains, and nuts as well as low intake of red and processed meats, refined grains, are popular in both Eastern and Western countries. Some earlier studies have reported the favorable associations between adherence to the healthy dietary patterns and risk of some non-communicable diseases, such as cardiovascular disease (45, 46). Moreover, adherence to the healthy dietary patterns has also been associated with reduced risk of several cancers (47–49). In the current study, we found a significant inverse relationship between healthy dietary patterns and the risk of glioma, although the results were heterogeneous. Our results are in agreement with some previous studies (19, 20), which demonstrated that adherence to the healthy dietary pattern was significantly associated with a reduced risk of glioma. There are several potential explanations for the beneficial effects of healthy dietary patterns on glioma risk. First, vegetables and fruits, two main components of healthy dietary patterns, are good sources of antioxidants, including vitamin C and E, polyphenol and other carotenoids compounds. Previous studies have clearly shown that these antioxidants can neutralize reactive oxygen species and protect against free radical damage involved in carcinogenesis (50, 51). Chen et al. also reported that oxidative stress, by producing reactive oxygen species, is involved in the pathogenesis of glioma (11). Second, vegetables and fruits contain high amounts of folate, which is necessary for synthesis of thymine and plays an important role in the synthesis, repair, and methylation of DNA, and thus preventing carcinogenesis (52). Third, fresh fish is rich in polyunsaturated fatty acids (PUFAs), especially omega-3 fatty acids, and thus may have anti-inflammatory properties and reducing the production of free radicals and carcinogens (53). Experimental studies have shown that above these compounds result in cell cycle arrest, and promote anti-tumor efficacies of lomustine in glioblastoma cells (54, 55). A recent meta-analysis examining the association of fish intake with glioma also concluded that higher consumption of fresh fish was associated with a reduced risk of glioma (56). Finally, vegetables, fruits and whole grains also contain large amounts of dietary fiber. Findings from previous studies showed that higher intake of dietary fiber could decrease inflammation, circulating estrogens and androgens and insulin resistance that play the important role in cancer prevention (57). Also, fermentation of fiber into short-chain fatty acids in the intestine may improve cells differentiation and apoptosis (18). Taken together, the aforementioned these studies support our results that healthy dietary patterns have the protective effect against glioma.

A high consumption of vegetables is one of the cornerstones of healthy dietary pattern, and has been recommended to the general public to reduce the risk of cancer (58). The results of this study showed a significant inverse association between vegetables intake and risk of glioma (RR = 0.84; 95% CI: 0.73–0.96; *P* = 0.012). Our results are in line with a previous meta-analysis conducted by Li and his colleagues (14), which indicated that intake of vegetables might have a protective effect on glioma. Similarly, previous meta-analyses have also demonstrated that a favorable effect was found between the intake of vegetables and risk for lung, breast and endometrial cancers (59–61). Several plausible explanations have been proposed for the protective effect of vegetables intake against glioma risk. First, vegetables consumed in healthy dietary patterns are good sources of antioxidants, e.g., vitamin C, vitamin E, and carotenoids. Earlier studies have shown that antioxidants may neutralize reactive oxygen species and reduce DNA damage (50, 51). Studies have shown that vitamin E abundant in green vegetables, can lower the expression of cyclin-dependent kinases 2,4 and over-expression of P27, through which it can protect brain cells (62). In addition, dark green leafy vegetables and orange vegetables are high in the carotenoids, which can turn into vitamin A (63). A previous meta-analysis of seven published articles showed that the highest category of dietary vitamin A was significantly associated with a reduced risk of glioma (RR = 0.80, 95% CI: 0.62–0.98) (64). Second, as mentioned previously, dark green vegetables are rich sources of folate. Davis and Uthus have demonstrated that folate is necessary for synthesis of thymine and plays an important role in the synthesis, repair, and methylation of DNA, and thus preventing carcinogenesis (52). Third, dietary fiber also found in vegetables, has been shown to play an important role in cancer prevention (57). Accordingly, the aforementioned these might account for the beneficial association between vegetables intake and glioma.

Meanwhile, findings from this meta-analysis also suggested that the intake of fruits might have a protective effect on glioma risk. Our findings were inconsistent with two previous meta-analyses, which showed that there were no significant association between the consumption of fruit and risk of glioma (8, 14). Compared with the present study, a recent systematic review and meta-analysis of dietary factors and risk of glioma concluded that total fruits intake had no significant effect on the risk of glioma (RR = 0.82, 95% CI: 0.59–1.12) (8). Similarly, results from a previous meta-analysis by Li also indicated no significant association between fruit intake and glioma (RR = 0.828, 95% CI: 0.659–1.039) (14). Discrepant findings might be originated from the inclusion of two new studies in our analyses. Moreover, we also excluded the two studies performed by Howe and Kaplan et al. which were done on brain tumors, while it was included in Li’ s meta-analysis (14). Likewise, our systematic review and meta-analysis has a larger sample size, which provides sufficient statistical power to detect the significance. However, current recommendations for fruits intake by the World Health Organization, World Cancer Research Fund, and American Institute for Cancer Research are important for cancer prevention (65). Several mechanisms support the observed favorable effect of fruits intake and risk of glioma, although the mechanisms have not been thoroughly investigated. First, the protective association of fruit intake against glioma might be explained by the high content of vitamin C. It is widely known that vitamin C can protect cells from oxidative DNA damage and inhibit the formation of *N*-nitroso compounds, thereby blocking carcinogenesis (17). In addition, a previous meta-analysis on vitamin C intake and glioma risk also showed that a higher intake of vitamin C was associated with a 14% reduced risk of glioma (pooled RR = 0.86; 95% CI: 0.75–0.99) (66). Second, fruit contains high amounts of dietary fiber, phenols, carotenoids, antioxidants, and flavonoids, the protective association of these constituents with glioma have been reported in previous studies (11, 14, 50, 51). As mentioned above, antioxidants from β-carotene and lycopene may neutralize reactive oxygen species and reduce DNA damage (51). Moreover, high fiber intake has been reported to play a key role in the prevention of glioma (14). Finally, fruit is also major source of folate. Experimental studies have illustrated that folate metabolism plays a key role in carcinogenesis, due to its involvement in DNA synthesis, methylation and repair (67). In a word, the aforementioned these studies support our results that higher fruit intake has a protective effect against glioma.

Although several previous meta-analyses have discussed the effect of fish intake on glioma risk (8, 56, 68), unfortunately, the results have been inconclusive. For example, a previous meta-analysis of eight observational studies, reporting the association between dietary fresh fish and processed fish intake and the risk of glioma, revealed that higher intake of fresh fish could significantly reduce the risk of glioma (RR = 0.823, 95% CI: 0.698–0.970) (68). Significant inverse association was also found in another meta-analysis by Lei et al. (56). Lei et al.’s study found that compared with the lowest category, the highest category of fresh fish intake was significantly associated with a reduced risk of glioma (OR = 0.72; 95% CI: 0.53–0.97; *P* = 0.032). By contrast, however, findings from a recent systematic review and meta-analysis of dietary factors and risk of glioma showed that fresh fish intake had no significant effect on the risk of glioma (RR = 0.86, 95% CI: 0.70–1.06) (8). Similar to the recent meta-analysis mentioned above, our study also found no significant association between fresh fish intake and risk of glioma. As previously mentioned, PUFAs and vitamin E abundant in fresh fish could affect oxidative stress, thereby reducing the production of free radicals and carcinogens (53). Even so, there may be several possible explanations for the observed null results. First, the apparent null result for fresh fish and glioma risk may be due to limited evidence available. The number of articles was relatively small, and only four articles (reporting five studies) published in English were included in this meta-analysis, which might omit other languages studies. What is more, we excluded three studies that had been included in previous meta-analyses (56, 68) but involved meningiomas and other types of brain tumors. Second, all the included studies were case-control studies, which might be subject to recall and selection biases. Third, in included studies, different level of fresh fish intake might be considered in the interpretation of null findings. Finally, the discrepant findings might also be explained by lack of controlling for several known confounders, e.g., exposure to high-dose ionizing radiation. In short, more high-quality and prospective cohort studies are required to ascertain the relationship between fresh fish intake and glioma risk.

In our analyses, it is also worth noting that no associations were found between the intakes of nuts, whole grains and dairy products and the risk of glioma (*P* > 0.05). Inconsistent with our findings, Malmir et al. found an inverse association between legume and nuts consumption and odds of glioma (69). The lack of significant associations for glioma may be due to the small number of available studies in our meta-analyses. Given these null findings, it is necessary to obtain more detailed information on these foods intake in future studies.

In the present meta-analysis, we found the high between-study heterogeneity on the association between adherence to the healthy dietary patterns and risk of glioma (*I*^2^ = 86.9%; *P*_for heterogeneity_ < 0.0001). Although between-study heterogeneity is common in meta-analysis (14), exploring the potential sources of heterogeneity is the essential. Hence, we performed subgroup analyses based on study design, study quality, country, and methods used to determine dietary patterns to explore sources of heterogeneity. The results showed that heterogeneity of healthy dietary patterns might be mainly due to the difference in study design, methods used to determine dietary patterns and comparison. There are several possible explanations for this high heterogeneity. First, nine of the included studies were case-control studies. Recall bias resulting from dietary survey methods (i.e., FFQs) in the case-control studies should be considered. In addition, there were only three cohort studies, which limited the significance of the combined results to a certain extent. Second, different models used to control potential confounding variables in included studies may explain the heterogeneity observed in our analyses. There was an inconsistent adjustment for potential confounding variables in the included studies. As a result, it is inevitable that we have high levels of heterogeneity when combining studies. Third, the heterogeneity is more evident in the results regarding the healthy dietary patterns, probably due to the difficulty in characterizing this pattern across the selected studies (70). Finally, the substantial heterogeneity would exist because the score range was divided into different intervals in different studies during the statistic analysis. The heterogeneity of vegetables intake mainly came from the difference in the study quality and design. In the subgroup of the study quality, the heterogeneity decreased from 73.5% to 0. Meanwhile, the articles with a case-control design had 24.0% heterogeneity after separating. For fruits intake, subgroup analysis revealed that the heterogeneity might be mainly due to the difference in study design. However, the considerable heterogeneity persisted in subgroup analyses, indicating the presence of other unknown confounding factors. Glioma is a complex etiology and pathophysiology disease generated by the combined effects of genetic and environmental factors. Thus, other genetic and environmental variables, as well as their possible interactions, may well be potential contributors to the heterogeneity observed. Finally, the heterogeneity of fresh fish, nuts, whole grains, and dairy products was small. Due to the limited number of included studies, we could not perform a subgroup analysis.

### Strengths and limitations

This study has its own strengths. First, this is the first systematic review and meta-analysis to comprehensively clarify the associations between healthy dietary patterns, typical healthy foods and the risk of glioma. This systematic review further adds to the current evidence on the protective effect of healthy dietary patterns against glioma. Second, the cases of glioma have been diagnosed through clinical manifestations and pathological section, avoiding misdiagnosis. Third, with a larger number of studies and the cases of glioma than the previous meta-analyses, this study had more statistical power to detect the significant associations between healthy dietary patterns, typical healthy foods and glioma. Fourth, the strengths of this study are the extensive literature screening with strict adherence to quality standard set out in PRISMA 2020 guidelines. We excluded some published studies that have been included in some previous meta-analyses but involved astrocytoma and other types of brain tumors (8, 14, 68). Fifth, subgroup and sensitivity analyses were performed to further detect the potential sources of heterogeneity, thereby improving the accuracy of the research results. Despite these strengths, several limitations should be noted when interpreting our findings. First of all, because of the observational nature of included studies, the recall and selection bias couldn’t be completely eliminated. Moreover, in the vast majority of included studies, dietary intake was assessed by self- reported FFQs, which carried an inherent recall bias. Thus, future large prospective studies and randomized controlled trials are needed to provide more robust evidence for the exact associations between whole dietary patterns and glioma. Second, there was evidence of between-study heterogeneity in the main analysis. Subgroup analyses revealed that the differences in the sample population, methods used to define healthy dietary pattern, and the limited number of included studies might have contributed to the observed heterogeneity. Third, there was a significant publication bias in the healthy dietary patterns. Even after the trim and fill analysis was conducted, the results did not change considerably. Fourth, although the majority of the included studies adjusted for some potential confounders, residual and unmeasured confounding effects cannot be ignored. Fifth, the small number of available studies did not allow us to consider the effects of distinct dietary patterns as well as to analyze sex-disaggregated data. Finally, the present study had a geographical restriction, as the most studies on the healthy dietary patterns and glioma have been performed in Middle Eastern countries, where the dietary intakes are markedly different from the Western countries. Accordingly, more research, particularly in European or developed countries, is needed to confirm our findings.

## Conclusion

In conclusion, the present systematic review and meta-analysis shows that adherence to healthy dietary patterns may reduce the risk of glioma. Moreover, higher intakes of several typical healthy foods, such as fruits and vegetables are also inversely associated with the risk of glioma. Our findings provide a more comprehensive and reliable evidence for the protective role of healthy dietary patterns, typical healthy foods against glioma. This supports public health recommendations to increase the intakes of healthy dietary patterns and typical healthy foods for the prevention of glioma. However, further large prospective studies and randomized controlled trials are required to validate our findings in different populations.

## Data availability statement

The original contributions presented in this study are included in the article/Supplementary material, further inquiries can be directed to the corresponding author.

## Author contributions

LS and FJ took responsibility for data integrity and the accuracy of data analysis and drafted the manuscript. FJ was responsible for study concept and design. LS and DY acquired the data. LS was responsible for analysis and interpretation of the data. DY performed the statistical analysis. All authors critically revised the manuscript for important intellectual content and approved the submitted version.
