# Supplementary figures and images for: Healthy dietary patterns, foods, and risk of glioma: A systematic review and meta-analysis of observational studies
Source: Front Nutr. 2023 Jan 4;9:1077452. doi: 10.3389/fnut.2022.1077452 (PMC9845718; doi:10.3389/fnut.2022.1077452)

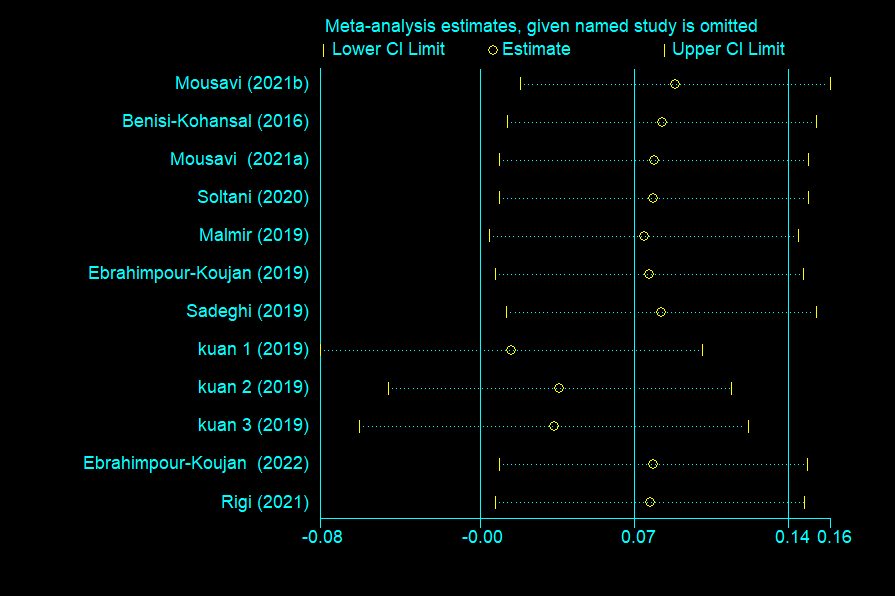

Supplement: Supplementary file 1 [file Image_1.TIF]
